# Network-meta analysis made easy: detection of inconsistency using factorial analysis-of-variance models

**DOI:** 10.1186/1471-2288-14-61

**Published:** 2014-05-10

**Authors:** Hans-Peter Piepho

**Affiliations:** 1 Bioinformatics Unit, Institute of Crop Science, University of Hohenheim, Fruwirthstrasse 23, 70599 Stuttgart, Germany

**Keywords:** Analysis of variance, Baseline contrast, Heterogeneity, Inconsistency, Linear mixed model, Network meta-analysis, Pairwise treatment contrast, PRESS residual, Studentized residual

## Abstract

**Background:**

Network meta-analysis can be used to combine results from several randomized trials involving more than two treatments. Potential inconsistency among different types of trial (designs) differing in the set of treatments tested is a major challenge, and application of procedures for detecting and locating inconsistency in trial networks is a key step in the conduct of such analyses.

**Methods:**

Network meta-analysis can be very conveniently performed using factorial analysis-of-variance methods. Inconsistency can be scrutinized by inspecting the design × treatment interaction. This approach is in many ways simpler to implement than the more common approach of using treatment-versus-control contrasts.

**Results:**

We show that standard regression diagnostics available in common linear mixed model packages can be used to detect and locate inconsistency in trial networks. Moreover, a suitable definition of factors and effects allows devising significance tests for inconsistency.

**Conclusion:**

Factorial analysis of variance provides a convenient framework for conducting network meta-analysis, including diagnostic checks for inconsistency.

## Background

Results from several randomized trials can be combined by meta-analysis methods. In the simplest case, all trials comprise the same set of treatments, typically only two, i.e., a new treatment and a control or baseline treatment. When trials differ in design, i.e., in the sets of treatments tested, joint analysis may be done by what has come to be called network meta-analysis (NMA). Such analyses combine different sources of pairwise treatment comparisons across trials, i.e., direct comparisons from trials that jointly test both treatments of interest and indirect comparisons from trials that only test one of the two treatments, but are connected through other treatments via the trial network. A key assumption of many methods for NMA is consistency of treatment effect estimates across designs, defined by the set of treatments tested. In particular, consistency implies agreement between direct and indirect evidence on a treatment contrast. Several methods have been proposed for detecting inconsistency in trial networks [1-4].

Most methods for analysis of NMA operate on pairwise contrasts of treatments with a baseline treatment or control, henceforth denoted as baseline contrasts. Some methods for detecting inconsistency in meta-analysis networks based on baseline contrasts are relatively complex on account of the fact that baseline treatments may vary among trials and sources of inconsistency have to be traced through loops of the network [3-5]. It has been shown by Piepho et al. [6] that NMA can be greatly simplified by modelling treatment means rather than treatment contrasts using factorial analysis-of-variance (ANOVA) models, and that such analyses can produce identical or essentially the same results as analyses using baseline contrasts. The present paper will therefore focus on the ANOVA approach and illustrate its versatility. Specifically, we will explore ways to detect inconsistency using standard procedures for linear models available in most statistical packages. The methods will be illustrated using the diabetes example published by Senn et al. [7]. This example has also been used by Krahn et al. [1] to illustrate their proposed methods for detection of inconsistency using a baseline contrast parameterization, so our results can be compared directly to that paper in order to appreciate the degree of agreement between both model formulations and the resulting tests and diagnostic checks for inconsistency. The presentation assumes that the reader has access to a mixed model package using restricted maximum likelihood (REML) to estimate variance components and is familiar with the essentials of the underlying theory [8]. Program code in SAS for all analyses presented is given in Additional file 1.

## Methods

In this section, we describe the basic models we are using. In the Results section, minor extensions and associated statistics derived from the various models, such as influence diagnostics, are introduced as needed.

A two-way ANOVA model for meta-analysis can be written as

(1a) $$\eta_{\mathrm{ij}}=\beta_{i}+\gamma_{j}+u_{\mathrm{ij}}$$

where *η*_*ij*_ is the expected value of the *j*-th treatment in the *i*-th trial, *β*_*i*_ is the main effect of the *i*-th trial, *γ*_*j*_ is the main effect of the *j*-th treatment, and *u*_*ij*_ is a trial × treatment interaction effect, which models heterogeneity between trials. For implementation it is convenient to represent the linear model (1a) in symbolic notation that is akin to model syntax used in linear model packages. We here use a notation originally proposed by Wilkinson and Rogers [9], which has hence been used by many authors [10,11] and has also been implemented in some linear model packages. The factors used for representing the model are given in Table 1.

**Table 1:** Description of factors used for representing factorial ANOVA models for NMA

**Factor symbol**	**Factor description**
G	Group of trials, trial type, design
S	Study, trial
T	Treatment

The two-way ANOVA model (1) can be represented as [9]

(1b) S×T=S+T+S.T

where × is an operator for crossing two factors or model terms, S is a factor identifying the individual trial, and T denotes the treatment factor. Effects in (1b) can be equated with those in (1a) as follows: S ≡ *β*_*i*_, T ≡ *γ*_*j*_, and S.T ≡ *u*_*ij*_.

In NMA, trials can be classified into groups of trials according to the set of treatments tested. These categories will henceforth be denoted as designs (Table 1). Procedures for detecting inconsistency can be easily implemented by expanding the ANOVA model (1) to reflect the nesting of trials within designs. The extended model is

(2a) $$\eta_{\mathrm{hij}}=\alpha_{h}+\beta_{\mathrm{hi}}+\gamma_{j}+v_{\mathrm{hj}}+u_{\mathrm{hij}}$$

where *α*_*h*_ is a main effect for the *h*-th design, *β*_*hi*_ is an effect for the *i*-th trial nested within the *h*-th design, *γ*_*j*_ is the main effect of the *j*-th treatment, *v*_*hj*_ is an interaction effect for the *h*-th design and the *j*-th treatment, and *u*_*hij*_ is an interaction effect for the *i*-th trial (nested within the *h*-th design) and the *j*-th treatment. The interaction effect *v*_*hj*_ represents inconsistency, whereas *u*_*hij*_ represents heterogeneity as in model (1) [6,12]. Using the factor G to represent the design (Table 1), the symbolic version of the extended model (2a) is

(2b) $$\left(G/S\right)\times T=G+G.S+T+G.T+G.S.T$$

where / is a nesting operator [9]. Note that the nesting relation G/S in (2b) is resolved as G/S = G + G.S. This structure is then fully crossed with T, as indicated by the crossing operator × on the left-hand side of eq. (2b). Effects in (2a) and (2b) can be equated as follows: G ≡ *α*_*h*_, G.S ≡ *β*_*hi*_, T ≡ *γ*_*j*_, G.T ≡ *v*_*hj*_, and G.S.T ≡ *u*_*hij*_.

Linear predictors (1) and (2) can be used either in models for individual patient data or in models for treatment summaries per trial (e.g., empirical logits or treatment means) [6]. When individual patient data are modelled, then depending on the outcome variable it may be appropriate to use the linear predictor in a generalized linear (mixed) model [GL(M)M], e.g., when the response is binomial so that a logit or probit link is required. When summary measures are available, it is customary to model the response by a linear (mixed) model assuming normality and accounting for possible heterogeneity in precision by weighting. In the diabetes example by Senn et al. [7], we have at our disposal mean responses per treatment and trial as well as the associated sample standard deviations and sample sizes, from which the variance of a mean can be computed. Thus, the models used for our analyses are of the form

(3) y=η+e

where *y* is the observed treatment mean in a trial, *η* is the linear predictor, modelled, e.g., using (1) or (2), and *e* is the random normal error associated with the observed mean. The errors are modelled to have zero mean and variance var(*e*) equal to the observed squared standard error of a mean, assumed to be a known constant when fitting (3). This analysis is easily performed using linear mixed model software with weighting facility by defining the inverse of var(*e*) as weight and fixing the residual variance at unity [13]. The approach is fully efficient, because the variance-covariance matrix of the vector of means *y* is diagonal with elements equal to var(*e*) [14].

Following Krahn et al. [1], we will initially consider analyses by models (1) and (2) when all effects in the linear predictor (eq. 1b or 2b) are taken as fixed. Subsequently, we will consider analyses that model heterogeneity [i.e., the interaction effects S.T and G.S.T in eqs. (1b) and (2b), respectively] as random, which is common practice (see, e.g., [6] and [15]). One may argue that if heterogeneity is detected, then the effect for heterogeneity may be used as an error term for testing inconsistency because heterogeneity effects are nested within the effects for inconsistency. This leads to an analysis with random interaction effects S.T or G.S.T. Conversely, one may insist that heterogeneity be modelled as a fixed effect. Then if heterogeneity is detected, it may be concluded that there is no further basis for testing inconsistency because of the nested structure of effects for heterogeneity in relation to inconsistency. In this situation, one may try to find subsets of trials that do not display heterogeneity and analyse these subsets separately [2]. This philosophy is in agreement with that put forward by Nelder [16], who argued that testing main effects in a two-way fixed-effects ANOVA is justified only when the interaction is deemed to be absent and the model is reduced accordingly. Here, we will present results for both approaches (interactions for heterogeneity fixed or random) and compare the results. Our favoured approach is to model heterogeneity as random when performing checks and tests for inconsistency as well as when comparing treatment means.

## Results

The diabetes data comprise a total of 26 trials, most of which involve a glucose lowering agent added to a baseline sulfonylurea therapy. The continuous outcome variable is blood glucose change as measured by the marker HbA1c in patients with type two diabetes. There were fifteen different designs, including one three-armed trial and fourteen trials involving only two treatments. The network provides direct evidence for fifteen out of 45 possible pairwise contrasts. Eight of these contrasts involve the placebo. The ten different treatments are given in Table 2.

**Table 2:** **Ten treatment groups of the diabetes example of Senn et al.**[7]

**Four-letter abbreviation of treatment**	**Treatment**
acar	Acarbose
benf	Benfluorex
metf	Metformin
migl	Miglitol
piog	Pioglitazone
plac	Placebo
rosi	Rosiglitazone
sita	Sitagliptin
SUal	Sulfonylurea alone
vild	Vildagliptin

### Fitting models (1) and (2)

We start by fitting models (1) and (2) as purely fixed effects models, which is equivalent to the models used by Krahn et al. [1]. Generally, throughout the example, we adhere to the order of effects as stated in models (1) and (2) and use sequential (incremental) fitting of terms, corresponding to Type I hypotheses in linear model procedures of the SAS system, which is used for all analyses presented in this paper. There are five designs that have more than one trial and so allow testing for heterogeneity. Thus, we first fit model (1) separately to each of these designs. The resulting Wald-type chi-squared statistics for significance of the trial × treatment interaction (*u*_*ij*_), along with the associated *p*-values, are shown in Table 3. There is significant heterogeneity for four out of five designs.

**Table 3:** **Wald-type chi-squared tests for heterogeneity (*u***_***ij***_**)**

**Design**	**Wald statistic**	**Number of studies**	**Degrees of freedom**	***p*-value**
benf:plac	4.38	2	1	0.0363
metf:plac	42.16	3	2	<0.0001
migl:plac	6.45	3	2	0.0398
rosi:plac	21.27	6	5	0.0007
rosi:metf	0.19	2	1	0.6655

Tests are given for five designs represented by more than one trial in the diabetes example of Senn et al. [7].

An overall test for heterogeneity is obtained by fitting model (2). The trial × treatment interaction (G.S.T) yields a chi-squared statistic of 74.45 on 11 d.f. (*p* < 0.0001). This chi-squared statistic for overall heterogeneity is equal to the sum of the chi-squared statistics for heterogeneity for the five designs in Table 3. When dropping the effect G.T from the model, the Wald-test for the effect G.S.T becomes a joint test for inconsistency and heterogeneity. The chi-squared statistic for this test equals 96.98 on 18 d.f. (*p* < 0.0001), and it is equal to Generalized Cochran’s Q [1]. Further note that the model T + S + T.S produces the same overall Q of 96.98. At this point, we can conclude that there is significant heterogeneity.

All chi-squared statistics presented so far are identical to those in Table 3 of Krahn et al. [1], who used a model based on baseline contrasts. We also obtain their chi-squared statistic for inconsistency, when we fit G.S.T as fixed and test the effect G.T (chi-squared = 22.53, d.f. = 7, *p* = 0.0021). But we favor a mixed model analysis with random trial × treatment interaction (G.S.T), because we consider it the major error term for testing the design × treatment interaction (G.T), which assesses inconsistency. At the same time, the trial effect needs to be modelled as fixed in order to maintain equivalence with the baseline contrast approach [6,7]. When we take the interaction effect for heterogeneity (G.S.T) as random, assuming a constant variance for this effect, the chi-squared statistic for inconsistency (G.T) drops to 2.27. The REML estimate of the variance for heterogeneity is ${\hat{\sigma }}_{u}^{2}=0.06932$. Note that this estimate corresponds to half the variance for heterogeneity with the baseline contrast approach [6,15] (usually denoted as *τ*^2^). Since the test for inconsistency now involves an estimated variance component, we use the Kenward-Roger method for approximating the denominator d.f. of a Wald-type F-statistic [17]. We find *F* = 0.32 on 7 numerator and 11 denominator d.f. and *p* = 0.9268. By this analysis, there is no significant inconsistency, which is in contrast to the analysis with fixed effects for G.S.T. Note that this analysis treats the residual variances of the individual trials as known constants, although they are, in fact, estimated when analysing individual trials. The added uncertainty associated with these variance estimates could be accounted for by using the Kenward-Roger method in a single-stage analysis modelling individual patient data [14], but differences compared to the two-stage analysis employed here are expected to be small so long as the sample sizes per treatment and trial are large enough, as is usually the case.

A very simple further check for inconsistency is to fit both G.T and G.S.T as random. The best linear unbiased predictors (BLUPs) of the G.T effects give a direct indication which treatment × design combinations contribute most to the inconsistency. With the diabetes example, the variance component for G.T is estimated to be zero, so the BLUPs for all G.T effects are zero, which is in agreement with the non-significant Wald-test for inconsistency.

### Locating inconsistency by detachment of individual designs

Locating inconsistency in the network may be based on a detachment of an individual design from the others by a suitable model formulation that allows testing the contribution of that individual design to inconsistency in the network as well as the inconsistency that remains after detaching that design. Krahn et al. [1] showed how to code a detachment model for baseline contrasts. Here, we show how to implement this approach based on a straightforward extension of the factorial model (2).

To illustrate, consider the first design in the diabetes example, which has fifteen designs, coded by a factor G. We may define a new factor D1 for the first design, which has two levels, one for the first design and another common level for the remaining fourteen designs (Table 4). Obviously, factors D1 and G have a hierarchical relationship, with G nested in D1. Thus, the interaction effect G.T, which assesses inconsistency, may be partitioned as

(4) $$\left(D1/G\right).T=D1.T+D1.G.T$$

**Table 4:** **Definition of detachment factors for testing inconsistency [D *k *.T; *k *** **∈ (1,…,15)]**

**Design (factor G)**	**Design no. (*k*)**	**Factor for detachment**	**Level of factor for the fifteen designs**														
			**1**	**2**	**3**	**4**	**5**	**6**	**7**	**8**	**9**	**10**	**11**	**12**	**13**	**14**	**15**
acar:plac	1	D1	1	0	0	0	0	0	0	0	0	0	0	0	0	0	0
acar:SUal	2	D2	0	1	0	0	0	0	0	0	0	0	0	0	0	0	0
benf:plac^§^	3	-	-	-	-	-	-	-	-	-	-	-	-	-	-	-	-
metf:plac	4	D4	0	0	0	1	0	0	0	0	0	0	0	0	0	0	0
metf:acar:plac	5	D5	0	0	0	0	1	0	0	0	0	0	0	0	0	0	0
metf:SUal	6	D6	0	0	0	0	0	1	0	0	0	0	0	0	0	0	0
migl:plac^§^	7	-	-	-	-	-	-	-	-	-	-	-	-	-	-	-	-
piog:plac	8	D8	0	0	0	0	0	0	0	1	0	0	0	0	0	0	0
piog:metf	9	D9	0	0	0	0	0	0	0	0	1	0	0	0	0	0	0
piog:rosi	10	D10	0	0	0	0	0	0	0	0	0	1	0	0	0	0	0
rosi:plac	11	D11	0	0	0	0	0	0	0	0	0	0	1	0	0	0	0
rosi:metf	12	D12	0	0	0	0	0	0	0	0	0	0	0	1	0	0	0
rosi:SUal	13	D13	0	0	0	0	0	0	0	0	0	0	0	0	1	0	0
sita:plac^§^	14	-	-	-	-	-	-	-	-	-	-	-	-	-	-	-	-
vild:plac^§^	15	-	-	-	-	-	-	-	-	-	-	-	-	-	-	-	-

Factors are defined for eleven designs in the diabetes example of Senn et al. [7] (due to the network structure, the other four designs do not contribute to the inconsistency).

^§^These designs do not contribute to the overall inconsistency interaction design × treatment (G.T).

Fitting both effects on the right-hand side of (4) simultaneously, the effect D1.T captures the contribution of the first design to the overall design × treatment interaction, i.e., to overall inconsistency, while the remainder of the interaction/inconsistency after detachment of the first design is captured by the effect D1.G.T. Using the syntax of Wilkinson and Payne [9] and observing the nesting of factors D1, G and S, the full model can be developed as follows:

(5) $$\begin{array}{ll} \;\left(D1/G/S\right)\times T= & D1+D1.G+D1.G.S+T\\ & +D1.T+D1.G.T+D1.G.S.T \end{array}$$

The same partitioning can be done, in turn, for each of the other fourteen designs. The coding for factors D*k* [*k* ∈ (1,…,15)], where *k* indexes the designs, is shown in Table 4. Table 5 shows the results of analysis by model (5) for the eleven out of fifteen designs which contribute to the design × treatment interaction of the network. The analysis was done taking the interaction for heterogeneity (D1.G.S.T) either as fixed or random. Note that in case of a fixed effect for heterogeneity the Wald-type chi-squared statistics for D1.T and D1.G.T in (5) (see Table 5) add up to the chi-squared statistic for overall inconsistency (G.T) in (2) (chi-squared = 22.53), but not when heterogeneity is modelled as random. When the heterogeneity effect is modelled as fixed, there are five designs with a significant contribution to the inconsistency (Table 5). The strongest contribution comes from the design rosi:SUal, which was also detected by Krahn et al. [1] as being the major source of inconsistency. For this design, as well as for the design metf:SUal, the test of the remainder of the inconsistency (D*k*.G.T) is non-significant, suggesting that one of these designs could be removed to instate consistency. When heterogeneity is modelled as random, however, there is no indication of inconsistency for any of the designs.

**Table 5:** **Wald-type chi-squared tests for inconsistency using detachment factors [D *k *.T; *k *** **∈ (1,…,15)]**

**Design**	**Design no. ( *k *)**	**Number of studies**	**Degrees of freedom for D *k *.T**	**Effect D *k *.G.S.T fixed**				**Effect D *k *.G.S.T random**			
				**D *k *.T**		**D *k *.G.T**		**D *k *.T**		**D *k *.G.T**	
				**Wald statistic**	***p*-value**	**Wald statistic**	***p*-value**	**Wald statistic**	***p*-value**^**§**^	**Wald statistic**	***p*-value**^**§**^
acar:plac	1	1	1	0.09	0.7699	**22.45**	**0.0010**	0.02	0.8889	2.25	0.8782
acar:SUal	2	1	1	0.01	0.9091	**22.52**	**0.0010**	0.01	0.9430	2.26	0.8765
metf:plac	4	3	1	0.46	0.4976	**22.07**	**0.0012**	0.04	0.8379	2.22	0.8814
metf:acar:plac	5	1	2	0.15	0.9297	**22.39**	**0.0004**	0.07	0.9634	2.18	0.8129
metf:SUal	6	1	1	**15.02**	**0.0001**	7.52	0.2758	1.63	0.2343	0.92	0.9835
piog:plac	8	1	1	**5.28**	**0.0215**	**17.25**	**0.0084**	0.43	0.5299	1.96	0.9062
piog:metf	9	1	1	**5.40**	**0.0201**	**17.13**	**0.0088**	0.43	0.5318	1.94	0.9081
piog:rosi	10	1	1	0.05	0.8280	**22.49**	**0.0010**	0.01	0.9065	2.27	0.8751
rosi:plac	11	6	1	**6.24**	**0.0125**	**16.30**	**0.0122**	0.74	0.4112	1.87	0.9168
rosi:metf	12	2	1	0.01	0.9199	**22.52**	**0.0010**	0.01	0.9276	2.25	0.8795
rosi:SUal	13	1	1	**15.76**	**<0.0001**	6.77	0.3424	1.79	0.2146	0.66	0.9930

Tests are reported for eleven detached designs in the diabetes example of Senn et al. [7] (due to the network structure, the other four designs did not contribute to the inconsistency). The effect D*k*.G.S.T was taken either as fixed or as random. Tests significant at the 5% level are boldfaced.

^§^Based on Wald-type F-test with denominator d.f. computed by Kenward-Roger method [17].

### Using influence diagnostics for design × treatment means

In order to detect influential or outlying observations in the network, we use a two-stage approach. In the first stage, we compute design × treatment means using model (2). In the second stage, we fit an additive two-way model of the form G + T to the design × treatment means. From this analysis, we can obtain residual and influence diagnostics [18,19] by standard procedures with most linear mixed model packages. The key idea is that observations contributing substantially to inconsistency will display strong G.T interaction effects, which in turn will be captured by the residuals of the additive model G + T.

Three different options are considered for handling the effect for heterogeneity (G.S.T) in the first-stage analysis based on model (2): (i) taking it fixed, (ii) taking it random and (iii) dropping it. It turns out that with options (ii) and (iii), the treatment means of designs 3, 4, 7, 11 and 12 are correlated, meaning that weighting by the inverse of the squared standard errors is only an approximate method (note that the designs in question are precisely the ones represented by several trials). An exact analysis requires carrying the full variance-covariance matrix of design × treatment means forward and specifying this as the residual variance-covariance matrix of the model fitted at the second stage [14]. This is easily done in SAS using the REPEATED statement with the option TYPE = LIN(1). Note that option (iii) is in line with common practice when the baseline contrast formulation is used [1] and heterogeneity is deemed absent. But heterogeneity was found to be significant for the diabetes data, so one may argue that this effect should be in the model for checking consistency. If the effect is in the model and taken as fixed (option i), effectively all trials are given the same weight, whereas when the effect is dropped (option iii), each trial is weighted according to the variances of the means, which explains the differences in results. Both analyses are not fully satisfactory because heterogeneity is not appropriately taken into account. Taking heterogeneity as random (option ii) is common practice in meta-analysis [6,15], and this is also our preferred approach over option (i) for the reasons stated at the end of the Methods section.

To compute influence diagnostics in stage two, we here use the output generated by the INFLUENCE option to the MODEL statement of the MIXED procedure of SAS (Version 9.4). The PRESS residuals and studentized residuals are shown in Table 6. The PRESS residual for the *m*-th observation is the raw residual when the *m*-th observation has been deleted for estimating the fitted value. Large residuals indicate design × treatment combinations contributing substantially to the overall inconsistency. When in the first stage the effect for heterogeneity in model (2) (G.S.T) is modelled as fixed, or when the effect for heterogeneity is dropped, then designs 6 and 13 stand out as having conspicuously large studentized residuals relative to the standard normal distribution and relatively large PRESS residuals, which is in agreement with the tests in Table 5 and also with the results by Krahn et al. [1]. When heterogeneity is modelled as random, however, the studentized residuals of all designs are inconspicuous, which also agrees with our results in Table 5. The agreement of results based on PRESS residuals with the tests in Table 5 is expected because the D*k* factor essentially invokes a deletion operation that separates the effect of the *k*-th design from the rest, which is exactly the effect of PRESS residuals computed here (Table 6). It is noted that the residuals of two-treatment designs are equal in absolute value and of opposite sign, as expected. Observations with no residuals correspond to designs that do not contribute to the design × treatment interaction in the network.

**Table 6:** Studentized residuals and PRESS residuals

**Design**	**Observation**	**Treatment**	**Model for heterogeneity**					
			**G.S.T fixed**		**G.S.T random**		**G.S.T dropped**	
			**PRESS residual**	**Studentized residual**	**PRESS residual**	**Studentized residual**	**PRESS residual**	**Studentized residual**
1	1	acar	0.0545	0.2443	0.0785	0.1453	0.0642	0.2925
	2	plac	-0.0545	-0.2443	-0.0785	-0.1453	-0.0642	-0.2925
2	3	acar	-0.0234	-0.1022	0.0619	0.1056	-0.0259	-0.1142
	4	SUal	0.0234	0.1022	-0.0619	-0.1056	0.0259	0.1142
3	5	benf						
	6	plac	.	.	.	.	.	.
4	7	metf	0.0547	0.3026	-0.0781	-0.2282	-0.0814	-0.6783
	8	plac	-0.0547	-0.3026	0.0781	0.2282	0.0814	0.6783
5	9	acar	-0.0894	-0.2408	-0.1507	-0.2601	-0.1137	-0.3070
	10	metf	-0.0276	-0.0930	0.0036	0.0075	0.0060	0.0205
	11	plac	0.1359	0.3615	0.1193	0.2273	0.1057	0.2833
6	12	metf	0.6807	3.6726	0.6095	1.1614	0.6910	3.8755
	13	SUal	-0.6807	-3.6726	-0.6095	-1.1614	-0.6910	-3.8755
7	14	migl	.	.	.	.	.	.
	15	plac	.	.	.	.	.	.
8	16	piog	-0.4337	-2.5934	-0.2802	-0.5585	-0.3638	-2.2987
	17	plac	0.4337	2.5934	0.2802	0.5585	0.3638	2.2987
9	18	metf	-0.4719	-2.9147	-0.2927	-0.5779	-0.3467	-2.3246
	19	piog	0.4719	2.9147	0.2927	0.5779	0.3467	2.3246
10	20	piog	-0.1074	-0.5173	-0.0073	-0.0141	-0.0445	-0.2173
	21	rosi	0.1074	0.5173	0.0073	0.0141	0.0445	0.2173
11	22	plac	-0.2802	-1.9593	-0.2100	-0.6391	-0.3181	-2.4974
	23	rosi	0.2802	1.9593	0.2100	0.6391	0.3181	2.4974
12	24	metf	-0.1105	-0.5920	-0.0616	-0.1610	-0.0179	-0.1005
	25	rosi	0.1105	0.5920	0.0616	0.1610	0.0179	0.1005
13	26	rosi	-0.7077	-3.7022	-0.6733	-1.2693	-0.7424	-3.9701
	27	SUal	0.7077	3.7022	0.6733	1.2693	0.7424	3.9701
14	28	plac	.	.	.	.	.	.
	29	sita	.	.	.	.	.	.
15	30	plac	.	.	.	.	.	.
	31	vild	.	.	.	.	.	.

Residuals for diabetes example of Senn et al. [7] were obtained by fitting the model G + T to design × treatment means computed from model (2) with different assumptions regarding the effect for heterogeneity (G.S.T).

**Figure 1:**
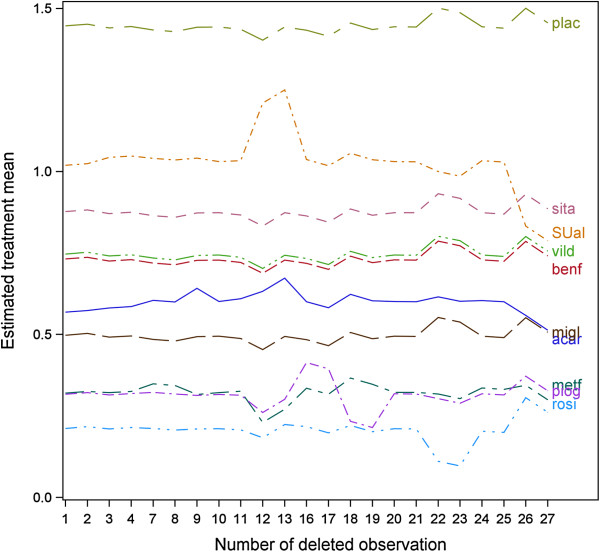
**Case-deletion plot of treatment means.** Case-deletion means based on a fit of the model G + T using design × treatment mean estimates obtained from fitting model (2) taking heterogeneity G.S.T as random. To obtain diagnostics for treatment means (factor T), we prevented an intercept from being fitted and imposed a sum-to-zero restriction on the design effects G.

A further set of useful diagnostic statistics are the case-deletion estimates of treatment means. Figure 1 shows a case-deletion plot for all treatment means against observations that contribute to the design × treatment interaction. The analysis is based on design × treatment means computed with random effects G.S.T in (2). The plot identifies the same observations as influential that also showed up by relatively large studentized and PRESS residuals in Table 6. For example, the treatment mean for SUal is largely driven by observations 12 and 13 from design 6 (metf:SUal) and observations 26 and 27 from design 13 (rosi:SUal). Also, the mean of treatment piog is mostly governed by observations 16 to 19 from designs 8 (piog:plac) and 9 (piog:metf).

### Presenting multiple comparisons of treatment means

Since the inconsistency has been found to be non-significant when modelling heterogeneity as a random effect, we drop the inconsistency interaction (design × treatment) from model (2) and then compute adjusted treatment means. We perform all pairwise comparisons using the simulation-based method of Edwards and Berry [20] at a family-wise significance level of 5%. Results are shown in Table 7. For ease of interpretation, we also compute a letter display using the algorithm described in Piepho [21]. According to the letter display, means sharing a common letter are not significantly different. It is seen that treatments acar, metf, migl, piog and rosi are significantly different from the placebo. Among these superior treatments, rosi has the smallest estimated mean, but this is not significantly different from the other treatments outperforming the placebo.

**Table 7:** Adjusted means for the ten treatments

**Treatment**	**Adjusted mean**	**Letter grouping**		
rosi	0.212			c
piog	0.317		b	c
metf	0.318		b	c
migl	0.496		b	c
acar	0.605		b	c
benf	0.709	a		c
vild	0.746	a		c
sita	0.876	a		c
SUal	1.029	a	b	
plac	1.446	a		

Means for the diabetes example of Senn et al. [7] were computed from model (2), dropping the design × treatment interaction (G.T) and modelling heterogeneity (G.S.T) as random. Pairwise comparisons at a family-wise Type I error rate of 5% by Edwards-Berry [20] test. Means with a common letter are not significantly different. The letter display was obtained by the method of Piepho [21]. Treatments are sorted in ascending order of means for ease of interpretation.

In order to emphasize that the ANOVA implementation also yields estimates of pairwise treatment contrasts and the associated standard errors, as does the baseline contrast implementation, we report these statistics in Table 8. This information is part of the standard output of mixed model packages, but is not convenient for routine reporting in case of a larger number of treatments. The table of treatment means and the associated letter display provide a more compact summary of the network meta-analysis.

**Table 8:** Pairwise differences of the ten treatment means

	**benf**	**metf**	**migl**	**piog**	**plac**	**rosi**	**sita**	**SUal**	**vild**
**acar**	-0.1045 (0.3659)	0.2870 (0.2504)	0.1085 (0.3280)	0.2880 (0.3054)	-0.8414 (0.2384)	0.3924 (0.2526)	-0.2714 (0.4165)	-0.4238 (0.2568)	-0.1414 (0.4159)
**benf**		0.3915 (0.3153)	0.2130 (0.3575)	0.3925 (0.3492)	-0.7369 (0.2776)	0.4968 (0.3038)	-0.1669 (0.4401)	-0.3194 (0.3622)	-0.0369 (0.4395)
**metf**			-0.1785 (0.2703)	0.0010 (0.2176)	-1.1284 (0.1494)	0.1053 (0.1600)	-0.5584 (0.3727)	-0.7109 (0.2272)	-0.4284 (0.3721)
**migl**				0.1795 (0.3093)	-0.9499 (0.2253)	0.2839 (0.2569)	-0.3799 (0.4091)	-0.5324 (0.3238)	-0.2499 (0.4085)
**piog**					-1.1294 (0.2119)	0.1043 (0.2163)	-0.5594 (0.4019)	-0.7119 (0.2914)	-0.4294 (0.4013)
**plac**						1.2337 (0.1235)	0.5700 (0.3414)	0.4175 (0.2326)	0.7000 (0.3408)
**rosi**							-0.6637 (0.3631)	-0.8162 (0.2290)	-0.5337 (0.3624)
**sita**								-0.1525 (0.4132)	0.1300 (0.4824)
**SUal**									0.2825 (0.4126)

Means for the diabetes example of Senn et al. [7] computed from model (2), dropping the design × treatment interaction (G.T) and modelling heterogeneity (G.S.T) as random. Table reports pairwise mean differences (and associated standard errors).

## Discussion and conclusions

This paper has illustrated how a factorial ANOVA approach can be used to perform NMA and to locate inconsistency in a given network. It was shown in Piepho et al. [6] that this analysis is either fully equivalent (summary measures, normal response in case of individual patient data) or very similar (individual patient data with non-normal responses and non-identity link functions in a GL(M)M framework) to the more commonly used approach to meta-analysis based on baseline contrasts. We think that the ANOVA approach has some practical advantages. Interpretation of results is facilitated by the focus on *t* treatment means rather than on *t*(*t* - 1)/2 pairwise contrasts. Standard procedures for multiple comparison of treatment means further aid the communication of results. Also, the approach may be appealing to those familiar with ANOVA of factorial experiments. It has been demonstrated that standard diagnostic procedures for linear models can be used to identify influential designs in the network and to detect sources of inconsistency. The results obtained for the diabetes example agree very closely with those obtained using recently proposed procedures based on a baseline-contrast approach [1]. We hope that this paper will help to popularize the ANOVA approach as a viable and easy-to-use approach to NMA.

## Competing interests

The author declares that there are no competing interests.

## Pre-publication history

The pre-publication history for this paper can be accessed here:

http://www.biomedcentral.com/1471-2288/14/61/prepub

## Supplementary Material

Additional file 1 Contains all SAS code that was used to obtain the results presented in this paper. Click here for file
